# Rural Health Workforce Development Through the AHEC Scholars Model

**DOI:** 10.1111/jrh.70193

**Published:** 2026-07-28

**Authors:** Danielle Bruno, Taylor O'Shaughnessy, Jennifer D. Taylor

**Affiliations:** ^1^ Department of Family Medicine, Indiana University School of Medicine Indianapolis Indiana USA

## Abstract

**Introduction:**

It is widely understood that access to health care services is crucial to achieving and maintaining good health. The Indiana AHEC Scholars program was developed to help combat these shortages by providing post‐secondary health professions students with a didactic curriculum paired with community‐based rural and/or medically underserved experiences in local communities. This study aimed to assess any relationship between students’ engagement in the AHEC Scholars program and both their intent to and actual practice in a rural community post‐graduation.

**Methods:**

We conducted a retrospective cohort study on five cohorts of post‐secondary health professions students who engaged in the AHEC Scholars program between 2018 and 2023. We conducted a quantitative analysis using a pre‐mid‐and post‐program evaluation. A paired *t*‐test was conducted to assess a change between a student's self‐reported intent to practice in a rural community at the start of the program compared to the midway point of the program and the end of the program. Additionally, a chi‐square test was conducted to assess the relationship between the students’ reported intent to practice in a rural area at the end of the Scholars program and their reported practice location 1 year post‐program.

**Results:**

A statistically significant difference was found between the students’ intent to practice in a rural setting before and after their involvement in the AHEC Scholars program with the post‐experience scores being significantly higher than the pre‐experience scores (*p* ≤ 0.01). Additionally, we found a significant change over time, as the students’ reported interest in rural practice increased between the start of the program and the mid‐point, (*p* ≤ 0.01), as well as a between the midpoint of the program and the end (*p* ≤ 0.01). Using a scale wherein 1 is low and 5 is high, the mean score for students’ intent to practice in a rural area between the pretest, mid‐point, and posttest evaluation were 3.02, 3.46, and 3.67, respectively. A significant correlation was also found between the students’ intent to practice in a rural area and actual rural practice, *p* ≤ 0.001. At the time of 1‐year follow‐up, 24.6% (*n* = 92) of individuals reported employment in a rural area.

**Conclusion:**

Our study highlighted that incorporating didactic and community‐based experiences may contribute to health profession education students choosing to practice in rural communities when entering the workforce. Our findings demonstrate the vital need to develop clear career trajectories for health professional students to learn about rural‐based care as a strategy for enticing practice in those communities.

## 1 Introduction

Access to health care services is crucial to achieving and maintaining good health, yet residents living in rural areas frequently encounter barriers to those critical health care services, limiting their ability to identify and obtain the care that they need. One significant barrier is workforce shortages, as they restrict access to health care by limiting the supply of available services [1]. As of 2025, approximately 66.40% of Primary Care Health Professional Shortage Areas were in rural areas, meaning that 77,253,848 people living in these areas face difficulty accessing adequate health care services [2]. Simply stated, approximately 20% of the US population live in rural areas yet only 10% of the physician workforce practice in rural areas [3]. While research has indicated that the density of primary care clinicians (physicians, nurse practitioners, physician assistants) in rural areas is increasing, the pace is not keeping up with the increase in urban areas [4]. Continued work is necessary to develop a strong pathway into rural‐based health care careers to improve access to primary health care services in rural communities [3].

In response to health workforce shortages in rural areas, a variety of programs and incentives have been implemented in an effort to recruit and retain a health workforce in rural and underserved areas [3]. Once such program, the Area Health Education Centers (AHEC) program, was developed by the US Congress to recruit, train, and retain a health professional workforce committed to serving rural and underserved populations [5]. AHECs are specifically designed to develop and enhance education and training networks within communities, academic institutions, and community‐based organizations, and as a result, broaden the distribution of the health workforce, improve health care quality, and improve health care in rural and other high‐need areas [6]. As a strategy to address health workforce shortages, AHECs were charged by the US Health Resources and Services Administration (HRSA) to develop and implement the AHEC Scholars program, a longitudinal, interdisciplinary program curricula that implements a defined set of clinical, didactic, and community‐based training activities in rural and/or underserved urban areas for health professions students [6].

The Indiana AHEC Scholars program was designed for post‐secondary health professions students, in the last 2 years of their academic training, interested in supplementing their education by gaining additional knowledge and experience in rural and/or underserved urban settings. Initiated in 2018, the 2‐year program utilized an interdisciplinary didactic curriculum coupled with a defined set of clinical and community‐based activities. The purpose of this side‐by‐side training was to help ignite or strengthen a student's interest in practicing in rural and underserved communities upon completion of their academic program. Students accepted into the Indiana AHEC Scholars Program were in the last 2 years of their health professions training program so that their academic graduation coincided with graduation from the program. Participants in the Indiana AHEC Scholars program completed 80 h of online didactic modules and 80 h of community‐based learning over 2 years. The curriculum highlighted competencies directly tied to rural health needs, including social determinants of health, interprofessional practice, behavioral health integration, practice transformation, and telehealth. These modules were reinforced through community‐based experiences in rural clinics, health fairs, and partnerships with local clinical and community‐based organizations. Existing literature report that multiple rural education initiatives and concluded that programs with structured didactic content, whether in the form of modules, seminars, or rural‐focused coursework, consistently increased students’ intentions to practice rurally and influenced actual workforce outcomes [4, 7]. Together, these studies confirm that didactic training combined with meaningful rural practice experiences is a key factor in preparing and retaining health professionals for rural communities. Previous research demonstrated a positive correlation between a health profession student's engagement in a rural‐based clinical rotation and self‐reported intent to practice in a rural community [8, 9, 10]. Given the focus on rural‐based health care within the AHEC Scholars program, we conducted a retrospective cohort study on five cohorts of students to assess the relationship between a student's engagement in the AHEC Scholars program, their intent to practice in a rural community, and actual practice in a rural location 1 year post‐graduation.

## 2 Methods

Health profession students in the last 2 years of their clinical training program engaged in 80 h of extra‐curricular didactic and 80 h of community‐based experiences across a 2‐year span. The didactic and community‐based experiences were designed to provide students with the opportunity to better learn about the nuances of health care in rural and medically underserved communities. The AHEC Scholars program was divided into block segments whereas every 2–3 months, scholars completed a set of specific asynchronous didactic modules in a Canvas learning platform system as well as engaged with their peers through planned community‐based experiences. The curriculum used a spiral methodology integrating the core topic areas in a manner to enhance learner knowledge and skill development. For example, the didactic subjects include “Interprofessional Teams” with a group discussion on teamwork in rural Indiana, “Advocacy and Community Partnerships” and “Strength‐Based Coaching and Innovative Care Models.” The community‐based experiences were designed to allow learners to better understand rural‐based health care and apply the knowledge they were gaining from the didactic modules.

During the program, students took four evaluations. A baseline evaluation was collected at the start of the program during orientation. Students repeated the evaluation survey at the halfway point of the program (end of year 1) and again at the end of the 2‐year program. Students received an email to complete a follow‐up survey 1 year post‐graduation. The program evaluation included questions around student confidence level in specific competencies related to the program such as identifying how an individual's family and social support system and other socioeconomic resources impact their health and health care or how to collaborate with health care organizations and other local health care agencies to improve patient care. The evaluation survey also asked students to rank their future practice plans, such as practicing in a rural community, on a Likert scale of one to five where one indicated strongly disagree and five indicated strongly agree. The evaluation survey was developed internally 8 years ago to assess the impact of the Indiana AHEC Scholars program, as existing instruments did not adequately capture its core competencies (e.g., interprofessional education, behavioral health integration, practice transformation, and more). The tool has been used consistently over 8 years and has demonstrated appropriate internal consistency (Cronbach's α = 0.62) and content validity through expert review. Although not formally published due to its program‐specific focus, its sustained use supports its reliability and suitability for this study.

The evaluation surveys were completed by students in the same Canvas learning platform system that housed their didactic modules. The 1‐year follow‐up survey was provided to the Scholars via email using a FormAssembly platform. The 1‐year follow‐up survey asked participants to share if they were pursuing further education, residency training, or employed. The survey asked individuals to provide the location of their employment and if they were practicing in a rural or medically underserved community. Individuals self‐reported the location of their employment setting as rural, primary care, or in a medically underserved community. Our study looked specifically at if the program resulted in an increase in intent and actual practice in rural communities.

To assess any change in student's intent to practice in a rural community, we conducted a paired *t*‐test to assess a change between a student's self‐reported intent to practice in a rural community at the start of the program compared to the midway point of the program and the end of the program. Additionally, we conducted a chi‐square test to assess the relationship between the students’ reported intent to practice in a rural area at the end of the Scholars program and their reported practice location for 1 year post‐program. We conducted independent sample *t*‐tests to assess any difference in results in rural practice based on self‐reported demographics (gender, growing up in either a rural or educationally/economically under‐resourced community. Additionally, a Kruskal–Wallis *H* test was performed to assess any difference in rural practice based on academic program. Using SPSS version 29, a two‐sided *p*‐value of less than 0.05 was statistical significance.

## 3 Results

Out of the 422 health profession students who complete the AHEC Scholars program between 2018 and 2024, 402 completed the 1‐year follow‐up for inclusion in the study, a 95.3% response rate. Among the students, 79% (*n* = 315) reported being female, 54% (*n* = 215) from a rural area, and 53% (*n* = 214) from an educationally or economically under‐resourced community. While in the AHEC Scholars program, students were studying a variety of health‐related academic programs including medicine (25%, 101), nursing (29% (121), pharmacy (10%, 41), and many more (Table 1).

**TABLE 1:** Scholars by self‐identified demographics.

	*N* = 402	%
Male	83	20.85%
Female	315	79.15%
Not reported	4	
American Indian or Alaska Native	1	1.00%
Asian	33	8.25%
Black or African American	44	11.00%
More than one race	15	3.75%
White or Caucasian	307	76.75%
Not reported	2	
Hispanic/Latino	23	5.74%
Non‐Hispanic/Latino	378	94.26%
Not reported	1	
Grew up in a rural area	215	54.43%
Grew up in an economically or educationally under‐resourced area	214	53.37%
**Academic program**		
Exercise science	5	1.24%
Medicine	90	22.39%
Nurse practitioner	21	5.22%
Pharmacy	41	10.20%
Physical therapy	20	4.98%
Physician assistant	25	6.22%
Psychology	4	1.00%
Public health	30	7.46%
Occupational therapy	10	2.49%
Registered nursing	94	23.38%
Social work	12	2.99%
Speech and language therapy	12	2.99%
Other	38	9.45%

**FIGURE 1:**
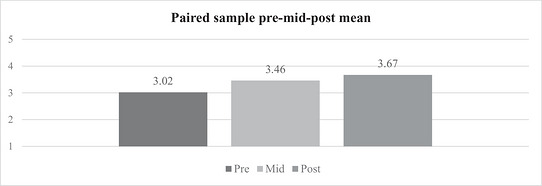
Paired sample mean (where 1 is low and 5 is high) on student's intent to practice in a rural area between the pretest, mid‐point, and posttest evaluation.

A paired *t*‐test was used to assess a change between a student's self‐reported intent to practice in a rural community at the start of the program compared to the end. The results for intent to practice in a rural community showed a statistically significant difference, *t*(382) = –10.002, *p* < 0.01, with the post‐experience scores being significantly higher than the pre‐experience scores. We further found that there was a significant change over time, whereas the there was a significant increase in students’ reported interest in rural practice between the start of the program and the mid‐point, *t*(397) = –8.139, *p* < 0.01, as well as a statistically significant change between the mid‐point of the program and the end, *t*(383) = –4.305, *p* < 0.01 (Figure 1). A chi‐square test of independence was performed to examine the correlation between intent to practice in a rural area and actual rural practice. The chi‐square test was statistically significant, *χ* ^2^(1, *N* = 361) = 20.379, *p* ≤ 0.001, indicating a significant relationship between reported intent and actual rural practice. In the 1‐year follow‐up, 24.6% (*n* = 92) individuals reported being employed in a rural area.

An independent *t*‐test found no differences in the results of the program based on gender, but we did find a difference on student's responses based on if they self‐reported growing up in an economically or educationally under‐resourced community. An independent *t*‐test found that students who reported growing up in an under resourced had a significantly higher rate of reporting an intent to practice in a rural area at the start of the AHEC Scholars program [*t*(394) = –3.371, *p* < 0.001], at the mid‐point of the program [*t*(394) = –3.229, *p* < 0.001] and with reporting practicing in a rural community 1 year later [*t*(371) = –3.481, *p* < 0.001], but was nearing significance at the conclusion of the program [*t*(385) = –1.920, *p* = 0.056]. An independent *t*‐test found that students from a rural area had a significantly higher rate of reporting an intent to practice in a rural area at the start of the AHEC Scholars program [*t*(388) = –5.411, *p* < 0.001], at the mid‐point of the program [*t*(388) = –4.908, *p* < 0.001], at the conclusion of the program [*t*(379) = –3.467, *p* < 0.001], and with reporting practicing in a rural community 1 year later [*t*(366) = –5.019, *p* < 0.001]. Table 2 outlines differences in participation in rural practice 1 year after program completion across demographic and background characteristics. Female participants were more likely than male participants to be practicing in a rural setting 1 year post‐program (*M* = 0.28 vs. *M* = 0.19). Participants who grew up in a rural area demonstrated a substantially higher likelihood of rural practice (*M* = 0.35, SD = 0.477) compared to those who did not grow up in a rural area (*M* = 0.13, SD = 0.333). Similarly, individuals who grew up in economically or educationally under‐resourced areas were more likely to be practicing rurally 1 year after program completion (*M* = 0.32, SD = 0.466) than those who did not have this background (*M* = 0.16, SD = 0.369). Overall, rural upbringing and experience in under‐resourced communities were associated with higher rates of rural practice 1 year following program participation.

**TABLE 2:** Differences between groupings on rural practice 1 year post‐program.

	*N*	Mean	SD
Male	80	0.19	0.393
Female	290	0.28	0.442
Grew up in a rural area	202	0.35	0.477
Did not grow up in a rural area	116	0.13	0.333
Grew up in an economically or educationally under‐resourced area	200	0.32	0.466
Did not grow up in an economically or educationally under‐resourced area	173	0.16	0.369

## 4 Discussion

Our study highlighted that incorporating didactic and community‐based experiences in health profession education results in a higher likelihood that they will choose to practice in rural communities when entering the workforce. Research shows that the combination of didactic learning and firsthand experience gives students a realistic view of rural practice and supports their long‐term interest in serving these communities [11]. The AHEC Scholars model demonstrates how structured didactic and community‐based experiences can influence students’ interest and eventual practice in a rural community [12, 13]. The results of the study demonstrated a steady increase in interest in rural‐based practice among participants in the program, likely based on the curriculum's focus on how to care for the unique needs of individuals in rural communities. The innovative nature of the AHEC Scholars program lies within the combination of its focused curriculum and provision of varied real‐life experiences in rural and underserved communities. By combining didactic education with community‐based application of knowledge, students can build the skills and self‐efficacy necessary to provide care to those living in rural and underserved communities.

Our findings align with similar programs that utilize rural‐heath seminars, community‐oriented projects, didactics, and community‐based experiences to encourage students to practice in rural communities [14, 15, 16, 17]. Similar studies on AHEC Scholars programs in other states demonstrate the positive shifts in health profession students’ knowledge and self‐efficacy as a result of their experiences [12, 13, 17, 18, 19]. Existing literature and our findings highlight a strong trend for the need for additional rural‐based didactic and rural‐based education across the health professions as a strategy to increase access to care in rural communities.

We had several limitations in the study that could impact the replicability of our study findings. The nature of the AHEC program focused on rural and underserved communities may have resulted in a section bias among students who applied to engage in the program. While all students engage in at least 80 h of extra‐curricular experiences across the 2 years of the program, they all have different experiences that could impact their interest in practicing in a rural care setting. Another limitation is that the medical students in the study would be entering residency, which is more typically located in urban areas and may have influenced the results of how many individuals were practicing in a rural area 1 year post‐program.

In a similar nature, our findings are based on that of the Indiana AHEC Scholars program, whereas similar AHEC Scholars programs in other states may not have the same result due to differing curriculum content and designs, as well as their rural/urban geographical makeup. We also recognize the possible courtesy bias that participants could have reported a more favorable response to rural‐based care given the intent of the program. Additionally, individuals self‐reported if they were employed in a rural community, which could have resulted in a discrepancy of the rural results based on self‐definition rather than the use of a formal RUCA code. Another limitation of the study is that while we had a strong sample size for a single program, the individual sample sizes among the different academic programs were too small to assess if the program results favor specific academic disciplines. Lastly, because this study only included data from the AHEC Scholars program, there was no control group for comparison. We feel that while the limitations are accurate, we mitigated some of the potential bias in the 1‐year follow‐up where we had a meaningful relationship between intent to practice and actual practice in a rural area.

We recognize that this is a point‐in‐time study and that our results may change if we conduct an additional survey with participants 5 years post‐graduation. While the findings of the study are promising, we continue to explore avenues to engage health profession training students in rural‐based education. For example, one challenge we have faced is that students need housing to engage in rural‐based clinical training due to the long distance between the students’ academic campus and a rural‐based clinic, yet rural communities face serious housing shortages that limit our ability to house students during rural clinical rotations. It is important to continue to engage with community partners within the rural communities to provide our students with many opportunities to better learn in and from rural communities.

## 5 Conclusion

Where you are from and where you train are two of the highest predictors of where you will practice [10, 20, 21]. With the ongoing health care workforce shortage in rural communities, it is vital that we develop clear career trajectories for health profession students to learn about rural‐based care as a strategy for enticing practice in rural communities. Our study demonstrated that incorporating didactic and community‐based experiences in health profession education resulted in a strong likelihood that the student will practice in rural communities when entering the workforce. Ideally, we will see those same individuals who completed the Scholars program in the past and now practicing in a rural community serve as clinical teachers for new Scholars in those same rural communities and create a strong cycle of developing a strong rural health workforce.

## AI Statement

During the preparation of this work the authors used ChatGPT to improve readability of the content. After using this tool/service, the authors reviewed and edited the content as needed and take full responsibility for the content of the publication.

## Funding

The contents of this article are solely the responsibility of the authors and do not necessarily represent the official views of the US Department of Health and Human Services, Health Resources and Services Administration, and Area Health Education Centers program. The project described was supported by Bureau of Health Professions, Health Resources and Services Administration Grant Number U77HP23068.

## Disclosure

The contents of this article are solely the responsibility of the authors and do not necessarily represent the official views of the US Department of Health and Human Services, Health Resources and Services Administration, Area Health Education Centers program.

## Ethnics Statement

This study was deemed “not human subject research” by the Institutional Review Board of Indiana University (protocol number 2006237989).

## Conflicts of Interest

The authors declare no conflicts of interest.
